# Spraying on a Summer Night: A Safer Way to Stop West Nile Virus

**Published:** 2004-08

**Authors:** Victoria McGovern

A population-level study has shown that night-time pesticide spraying in the late summer and early fall, aimed at controlling adult mosquitoes that carry West Nile virus, can be done in a way that does not drive up the number of people seeking emergency care for asthma-related problems **[*EHP* 112:1183–1187]**. A team led by Adam M. Karpati, a physician in the New York City Department of Health and Mental Hygiene, reports that in studies of the city’s 2000 mosquito spraying season, no correlation could be found between broad application of sumithrin (a pyrethroid pesticide) and asthma cases presenting at the city’s 11 public hospital emergency departments.

Earlier studies had shown that high exposure to pyrethroid pesticides—often in an occupational setting—can trigger reactions in asthma sufferers ranging from mild symptoms such as sneezing and scratchy throat to more acute ones such as wheezing, chest tightness, and even death. But no data have been available showing on a population scale how the lower-level exposures that come from public health spraying of pesticides affect the large number of asthmatics that may live in a big city.

The researchers tabulated data for asthma-related emergency room visits around the dates when a sumithrin-based pesticide was sprayed in each of 162 residential zip code areas in the city during July–September 2000. The timing of spraying within each zip code depended on whether surveillance indicated it was warranted—for example, if a dead bird were found to be infected with the virus, or if a human case were identified. A zip code area was rarely sprayed on consecutive days. The study also incorporated air quality data including daily measures of ozone, air particulates, and temperature, which can all cause fluctuations in the number of people seeking treatment for asthma-related symptoms. For a control, the team used asthma-related emergency room visits on days prior to spraying. They also looked at the number of asthma-related emergency room visits before and after the spraying season.

The researchers found that the number of asthma-related visits in the three days before application of the pesticide and the three days after were nearly identical. Looking more specifically within the emergency department data for asthma flare-ups in children and for aggravation of chronic obstructive pulmonary disease similarly yielded no correlation between spraying and symptoms.

The study does not necessarily show that public health pyrethroid spraying is not a danger to asthmatics. Rather, it could suggest that the city’s method of application and/or the citizens’ behavior during spraying helped minimize exposure. During 2000, the first year when New York City exclusively used a pyrethroid pesticide, the city limited its spraying to areas where the virus was detected in birds, mosquitoes, or humans, with spray trucks usually beginning their rounds near 10 p.m. and continuing through the night to 5 a.m. Radio, television, and print media were used to alert residents 48 hours prior to any spraying and to instruct people to remain indoors and close their windows during the hours when spraying would occur.

## Figures and Tables

**Figure:**
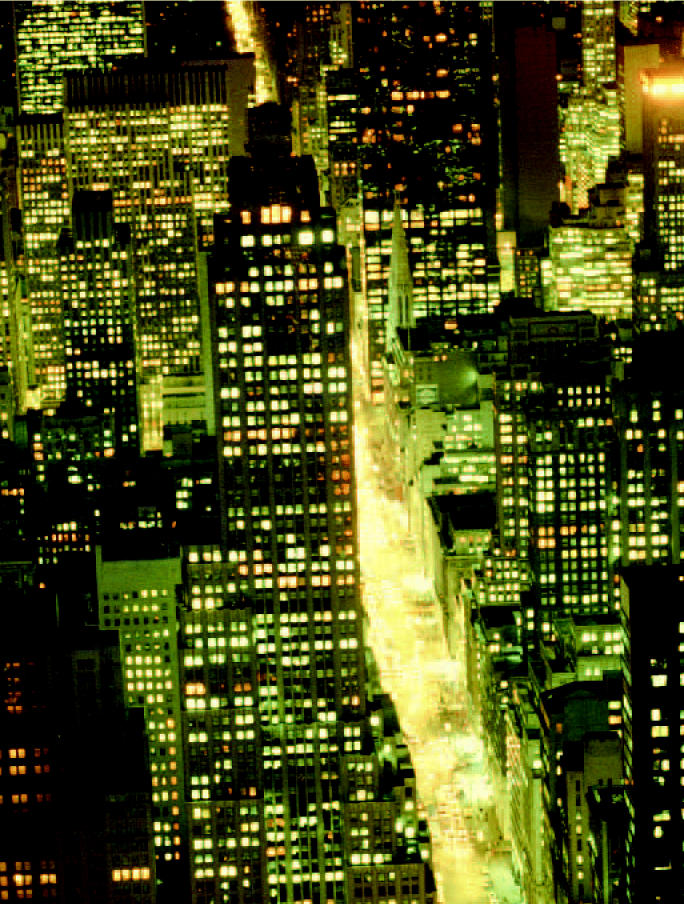
**Big Apple air okay.** Mosquito pesticide spraying to prevent West Nile virus was not associated with an increase in asthma attacks.

